# Anti-Cyclic Citrullinated Peptide (Anti-CCP) and Anti-Mutated Citrullinated Vimentin (Anti-MCV) Relation with Extra-Articular Manifestations in Rheumatoid Arthritis

**DOI:** 10.1155/2014/536050

**Published:** 2014-04-07

**Authors:** Laura Gonzalez-Lopez, Alberto Daniel Rocha-Muñoz, Manuel Ponce-Guarneros, Alejandra Flores-Chavez, Mario Salazar-Paramo, Arnulfo Nava, Ernesto German Cardona-Muñoz, Nicte Selene Fajardo-Robledo, Soraya Amali Zavaleta-Muñiz, Teresa Garcia-Cobian, Jorge Ivan Gamez-Nava

**Affiliations:** ^1^Department of Internal Medicine—Rheumatology, Hospital General Regional 110, IMSS, Avenida Salto del Agua 2192, Colonia Jardines del Country, 44710 Guadalajara, Jal, Mexico; ^2^Postdoctoral Program of the National Council of Science and Technology (CONACYT), 03940 Mexico City DF, Mexico; ^3^Postgraduate Programs of Pharmacology and Public Health Sciences, University Center of Health Sciences (CUCS), University of Guadalajara, 44340 Guadalajara, Jal, Mexico; ^4^Clinical epidemiology Research Unit, UMAE, Specialties Hospital, Western Medical Center, Mexican Institute for Social Security (IMSS), 44340 Guadalajara, Jal, Mexico; ^5^Physiology Department, CUCS, University of Guadalajara Research Division, UMAE, CMNO, IMSS, 44340 Guadalajara, Jal, Mexico; ^6^Department of Physiology, CUCS, University of Guadalajara, 44340 Guadalajara, Jal, Mexico; ^7^Postgraduate Program of Biomedical Sciences (Immunology), CUCS, University of Guadalajara, 44340 Guadalajara, Jal, Mexico

## Abstract

We evaluated the association between anti-cyclic citrullinated peptide antibodies (anti-CCP) and anti-mutated citrullinated vimentin antibodies (anti-MCV) with the presence of extra-articular (ExRA) manifestations in 225 patients with rheumatoid arthritis (RA). Ninety-five patients had ExRA and 130 had no ExRA. There was no association of anti-CCP and anti-MCV levels with the presence of ExRA as total group (*P* = 0.40 and *P* = 0.91, resp.). Making an analysis of individual manifestations, rheumatoid nodules were associated with positivity for rheumatoid factor (RF); (*P* = 0.01), anti-CCP (*P* = 0.048), and anti-MCV (*P* = 0.02). Instead, RF, anti-CCP, or anti-MCV were not associated with SS, chronic anemia, or peripheral neuropathy. Levels of anti-CCP correlated with the score of the Health Assessment Questionnaire-Disability Index (HAQ-Di) (*r* = 0.154, *P* = 0.03), erythrocyte sedimentation rate (ESR); (*r* = 0.155, *P* = 0.03), and RF (*P* = 0.254, *P* < 0.001), whereas anti-MCV titres only correlated with RF (*r* = 0.169, *P* = 0.02). On adjusted analysis, ExRA was associated with longer age (*P* = 0.015), longer disease duration (*P* = 0.007), higher DAS-28 score (*P* = 0.002), and higher HAQ-DI score (*P* = 0.007), but serum levels of anti-CCP and anti-MCV were not associated. These findings show the need to strengthen the evaluation of the pathogenic mechanisms implied in each specific ExRA manifestation.

## 1. Introduction

Extra-articular manifestations (ExRA) in patients with rheumatoid arthritis (RA) have been observed with at a frequency of 17.8 to 40.9% [1]. These manifestations may involve a multiplicity of organs and are of diverse severity [2]. ExRA are associated with comorbidities, erosions, more aggressive disease, high rate for disability, and premature mortality [3]. It has been described that patients with ExRA have a 2.5-fold increase in mortality compared with RA without ExRA [2]. Characteristics associated with ExRA include male gender, genetics (HLA-DRB1*04 subtype), positive rheumatoid factor (RF), antinuclear antibodies (ANA), and some environmental factors particularly smoking [4, 5].

Besides of RF and ANA, other autoantibodies have been tested as factors associated with ExRA. Anti-cyclic citrullinated peptide (anti-CCP) antibodies are commonly observed in the serum of RA patients, where the frequency varies between 55% and 69% [6]. Although several studies have evaluated the association of anti-CCP with ExRA, the results have not been always consistent. Turesson et al. in a case-control study found an association of rheumatoid factor (RF) and anti-CCP with the presence of ExRA, although these authors observed a borderline not significant difference in anti-CCP levels in ExRA compared with their controls without ExRA [7]. On the other side, Korkmaz et al. did not observe an association between anti-CCP with ExRA in patients with long disease duration or with early RA [8].

Antibodies against mutated citrullinated vimentin (anti-MCV) have been tested most recently for the diagnosis of RA, showing high sensitivity and specificity for the diagnosis of the disease [9]. Nevertheless, there is a lack of information on studies evaluating the association between anti-MCV with ExRA. To date, to the best of our knowledge, only one study has evaluated the possible association between anti-MCV antibodies with ExRA [10]. These authors did no observe an association between anti-MCV or anti-CCP with ExRA; however, this interesting study did not make explicit the methodology to identify ExRA making it necessary to reevaluate this information [10].

Therefore, because the information about the possible relationship between anti-MCV and ExRA is still insufficient, we designed a study to evaluate whether there is an association of anti-MCV or anti-CCP with ExRA.

## 2. Material and Methods

### 2.1. Study Population

We evaluated consecutive patients with RA from an outpatient secondary-care center in Guadalajara, Mexico (Department of Internal Medicine-Rheumatology, Hospital General Regional 110, IMSS). Study participants were included if they were Mexicans mestizo (defined as having at least two generations of ancestors born in Western Mexico) and only one person per family was recruited. To be included, patients with RA had to meet the ACR 1987 criteria for RA to have an established diagnosis and be 18 years old or older. Pregnant or nursing patients, those with other autoimmune disorders such as myasthenia gravis, Hashimoto tiroiditis, or any overlapping syndrome were excluded. Patients with diagnosis of chronic infections including B or C hepatitis, human immunodeficiency virus, tuberculosis, or other chronic infections were also excluded (the assessment for these exclusion criteria was based on the information obtained in a chart review of each patient).

### 2.2. Study Development

Patients were invited to participate and after signing an informed consent they were assessed by one researcher through a structured interview about epidemiological characteristics (such as age at the time of the study) and disease antecedents; disease duration was defined as the time from the onset of first symptoms of RA until the inclusion in the present study and was assessed through DAS-28 for disease activity, HAQ-DI for functioning, and other clinical measures.

All the included patients were systematically assessed during the evolution of the disease and at the time of the study for presence of eExRA (Table 1), using a structured protocol based on a modification of the criteria described by Turesson et al. [11] to identify patients with ExRA. Briefly, all the patients were systematically assessed by two researchers (both rheumatologists) with a structured interrogatory, physical examination, and chart review; if an ExRA was suspected, this patient was sent for confirmation to an specialist of the organ being involved (for example, ophthalmologist, cardiologist, pulmonologist, dermatologist, and nephrologist). The ExRA assessed included pericarditis (assessed by clinical judgment and confirmed by echocardiography), pleuritis (assessed by clinical judgment and thorax radiographs), Felty's syndrome (based on clinical evidence of splenomegaly confirmed by ultrasound and neutropenia <1.8 × 10^9^ described in at least 2 occasions), major cutaneous vasculitis (based on clinical judgment confirmed by biopsy), and neuropathy (based on clinical judgment and positive results poly/mononeuropathy at electromyography). Ocular involvement that was investigated included scleritis, epiescleritis, uveitis, or retinal vasculitis (these were diagnosed by ophthalmologists), glomerulonephritis (required being corroborated by nephrologist and renal biopsy if required), vasculitis involving other organs (these were identified by an specialist and if required a biopsy was performed), amyloidosis (based on clinical judgment and positive biopsy if required), keratonconjunctivitis sicca (was assessed in all patients and diagnosed if they had positive Rose-Bengal staining and positive Schirmer's test <5 mm/5 minutes), xerostomia (clinical judgment and abnormal sialometry and if required with minor salivary gland biopsy showing lymphocytic infiltrate), secondary Sjögren's syndrome (SS) (diagnosed if patients met at least two of the following criteria keratonconjunctivitis sicca, xerostomia, positivity for anti-Ro, or anti-La antibodies and lymphocytic infiltrate in minor salivary gland biopsy). Pulmonary involvement included bronchiolitis obliterans or organizing pneumonia (based on clinical judgment by a pulmonologist), whereas pulmonary fibrosis or interstitial lung disease were diagnosed based on clinical judgment by pulmonologist plus restrictive pattern in lung function test and confirmed by positive findings in high-resolution computed tomography of the lung. Cervical myelopathy was assessed by cervical radiographs showing increase in the atlantoaxial distance. Subcutaneous rheumatoid nodules were assessed in the physical examination and the diagnosis based in clinical judgment and biopsy if required. Chronic anemia was diagnosed if a hemoglobin <11 g/dL was observed at least in 3 occasions in the last 6 months before the evaluation and other causes of anemia were excluded. Severe manifestations were considered in presence of pulmonary fibrosis (or other entities involving lung), moderate or severe pericarditis/pleuritis, vasculitis involving major organs or cutaneous vasculitis with ulcers or gangrene, mono/polyneuritis multiplex, epiescleritis/scleritis, Felty's syndrome, amyloid deposition, and glomerulonephritis [1].

### 2.3. Determinations of Anti-MCV and Anti-CCP

At the same day of the clinical evaluation, a venous blood sample was obtained to quantify the titers of anti-CCP and anti-MCV antibodies. This sample was centrifuged and the serum was stored to −20°C until tested. Anti-CCP was detected with ELISA (DIASTAT, Axis-Shield Diagnostics Limited, UK). Positive anti-CCP was defined as a serum concentration ≥5 IU/mL. Sera with anti-CCP levels above the calibration curve were rerun after dilution to obtain actual semiquantitative values for all samples. ELISA kits for detection of anti-MCV (Orgentec Diagnostika GmbH, Mainz, Germany) were used according to manufacturer's instructions with the recommended cut-off value of 20 IU/mL.

### 2.4. Other Determinations

RF and C-reactive protein (CRP) were quantified in serum by nephelometry using a venous blood sample taken at the same day of the evaluation; the RF was measured on IU/mL and assessed using standard nephelometric assay according to the manufacturer's specifications (Dade Behring, DE). A positive result was defined as a level of >20 UI/mL. CRP was measured on mg/L and assessed using VITROS Chemistry systems CRP Slides, according to the manufacture's specifications (Ortho Clinical Diagnostics, INC.100.Indigo Creek Drive; Rochester NY 14626-5101).

Erythrocyte sedimentation rate (ESR) was determined using Wintrobe technique.

### 2.5. Statistical Analysis

Quantitative variables were expressed as means and standard deviations and qualitative variables in frequency and percentages. Comparisons in proportions between groups were performed with Chi-square (or Fisher exact test when required) and comparisons in means between groups were performed by Student's *t*-test for independent samples. A logistic regression analysis of factors associated with ExRA was performed and odds ratios (OR) and 95% confidence intervals (95% CI) were computed for each variable associated with ExRA introduced in the final model.

All analyses were performed two-tailed, and statistical significance was considered when *P* ≤ 0.05. All analyses were performed with SPSS software version 8.0.

### 2.6. Ethics

The study was approved by the Research and Ethics Committee of the Hospital in Guadalajara, Mexico. The study was approved by the Research and Ethics Committee of the Hospital Number of approval R-2010-1303-29. All participants signed a letter of voluntary informed consent. The study protocol followed the guidelines of the Helsinki declaration.

## 3. Results

**Figure 1:**
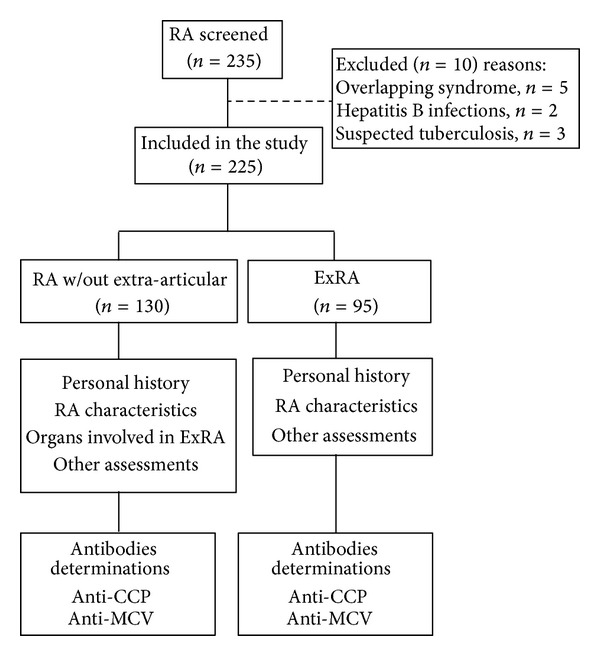
Study flow chart. RA: rheumatoid arthritis; ExRA: extra-articular manifestations; Anti-CCP: Anti-cyclic citrullinated peptide; anti-MCV: antimutated citrullinated vimentin.

Figure 1 represents the study flow chart. The total number of patients invited to participate was 235 with RA, of them 10 patients (4.3%) were excluded for the following reasons: five had overlapping syndrome, two had hepatitis B infection, and three were under study for tuberculosis.

Table 2 describes the clinical characteristics of 225 patients with RA included in the study. Most of them were women (92%), the mean age was 52.47 years, and 64 (28.4%) had a history of smoking. Regarding the characteristics of the disease, they had a mean for disease duration for 8.97 years, with a DAS28 score of 5.04 and HAQ-Di score of 0.84, whereas 94 (41.8%) had Steinbrocker radiological stage III or IV in their hands. Of these 225 patients, 95 (42.2%) had ExRA, the most frequently observed was SS in 25.8%, chronic anemia in 14.7%, rheumatoid nodules in 10.2%, and peripheral neuropathy in 5.3%. All the patients included had serological determination of anti-CCP and anti-MCV although only 204 patients had serum determination for RF. The frequencies of positivity for the autoantibodies were RF 66.2%, anti-CCP 68.9%, and anti-MCV 69.7%. The mean levels of RF were 119.11 IU/mL, of anti-CCP were 72.44 IU/mL, and of anti-MCV 156.85 IU/mL. One-hundred and forty patients (62.2%) had positivity for both anti-CCP and anti-MCV antibodies, while 110 patients (53.9%) displayed positivity for anti-CCP, anti-MCV and RF antibodies. Other characteristics of these 225 patients including laboratory variables and their treatments are also shown in this table.

Table 3 compares the clinical characteristics as well as the serological profile of RA patients without ExRA with those with ExRA. Patients with ExRA were older (*P* = 0.73) and had longer disease duration (*P* < 0.001), higher number of tender joints (*P* = 0.004), higher frequency of Steinbrocker radiological stage III or IV in their hands (*P* = 0.01), and higher levels of CRP (*P* = 0.01). No differences in titers of RF, anti-CCP, or anti-MCV were observed between patients with RA versus ExRA. Regarding the treatments, a higher proportion of patients without ExRA were receiving MTX at the time of the study compared with the group with ExRA (*P* = 0.007) although no statistical significant differences were observed between ExRA and the group without ExRA in MTX dose or anti-TNF agents.

In Table 4, we compare the frequency of specific ExRA of patients with positivity for these autoantibodies versus patients with negativity for these autoantibodies. Frequency of rheumatoid nodules was higher in patients with positive RF (14.1% versus 2.9%, *P* = 0.01), also positive anti-CCP higher frequency of rheumatoid nodules (12.9% versus 4.3%, *P* = 0.048). Similarly, the frequency of rheumatoid nodules was higher in patients with positive anti-MCV as compared with those without these antibodies (13.4% versus 2.9%, *P* = 0.02). Other specific manifestations including SS, chronic anemia, and peripheral neuropathy were not associated with the presence of positivity for these autoantibodies, whereas the number of patients with other specific manifestations such as pulmonary fibrosis, Raynaud syndrome, Felty syndrome, thrombosis, scleritis, or interstitial vasculitis were too low to allow statistical comparisons.

In data not shown in tables, no differences were observed in anti-CCP levels, in patients with SS versus patients without these manifestations (69.92 versus 80.15 IU/mL resp., *P* = 0.48), chronic anemia (72.97 versus 69.51 IU/mL resp., *P* = 0.84), rheumatoid nodules (69.80 versus 95.33 IU/mL resp., *P* = 0.18), or peripheral neuropathy (73.77 versus 45.57 IU/mL resp., *P* = 0.17). Similarly, no differences were observed in serum titers of anti-MCV in SS (160.86 versus 144.84 IU/mL resp., *P* = 0.57), chronic anemia (152.02 versus 184.71 IU/mL resp., *P* = 0.42), rheumatoid nodules (151.59 versus 201.06 IU/mL resp., *P* = 0.27), or peripheral neuropathy (158.92 versus 121.89 IU/mL resp., *P* = 0.57). In a subanalysis, patients with specific manifestations of ExRA were compared regarding to the number of positive autoantibodies that they had. Patients who had rheumatoid nodules had higher frequency of two or more types of autoantibodies compared with patients without nodules (90.9% versus 66.5% resp., *P* = 0.019). On the other side, the number of autoantibodies was not associated with SS (*P* = 0.8), chronic anemia (*P* = 0.5), or peripheral neuropathy (*P* = 0.4). Again total group patients with ExRA were not associated with the number of autoantibodies (*P* = 0.6).

Levels of anti-CCP correlated with the score of the Health Assessment Questionnaire-Disability Index (HAQ-Di) (*r* = 0.154, *P* = 0.03), erythrocyte sedimentation rate (ESR), (*r* = 0.155, *P* = 0.03), and RF (*P* = 0.254, *P* < 0.001), whereas anti-MCV titres only correlated with RF (*r* = 0.169, *P* = 0.02).

Table 5 shows a comparison of clinical characteristics between patients who presented a specific ExRA versus those who did not. Patients with SS had lower age (*P* = 0.001) and lower disease duration (*P* = 0.005), also had a higher proportion of patients diagnosed before year 2000 (*P* = 0.009), higher prevalence of Chloroquine treatment (*P* = 0.008), and lower prevalence of MTX treatment (*P* = 0.04). Instead, patients with anemia had a lower DAS28 (*P* = 0.005) and lower CRP levels (*P* = 0.015). In patients with rheumatoid nodules lower disease duration (*P* = 0.001), higher proportion of patients diagnosed before year 2000 (*P* = 0.010), higher proportion of Steinbrocker's score III or IV (*P* = 0.004), higher proportion of +RF (*P* = 0.01), higher frequency of +anti-CCP (*P* = 0.048), anti-MCV (*P* = 0.02), lower frequency of MTX utilization (*P* = 0.001), higher proportion of azathioprine users (*P* < 0.001) were observed.

Table 6 shows the results of the multivariate regression analysis of variables associated with ExRA presence. Using the forward stepwise method, variables associated with ExRA were age (*P* = 0.015), disease duration (*P* = 0.007), DAS-28 (*P* = 0.002), and HAQ-DI (*P* = 0.007). No significant associations were identified between presence of ExRA with anti-CCP and anti-MCV levels.

## 4. Discussion

Our results showed that 42% of our patients had ExRA; the variables associated with this involvement in the adjusted analysis are older age, longer disease duration, DAS-28 score, and HAQ-DI score, whereas no association was observed between anti-CCP or anti-MCV antibodies and ExRA as general group. For specific ExRA, there was a weak association between the presence of rheumatoid nodules and positivity for RF, anti-CCP, or anti-MCV antibodies but no relationship was found between these autoantibodies and SS, chronic anemia, or peripheral neuropathy.

The frequency of ExRA in RA observed in our study is similar to that described by Turesson et al. [12], where they observed that 40.6% of their patients with RA had ExRA. A review of 12 studies evaluating ExRA in RA revealed a variation of 17.8% to 40.9%; this frequency varies depending on the study designs, methodology, and definitions used for the detection of ExRA as well as characteristics of the center from where the patients were procured [1]. The most frequent ExRA observed in our study were SS, chronic anemia, and rheumatoid nodules. Among these different studies, the frequency of SS and Sicca syndrome vary from 7.3% to 19.6%, whereas the frequency of chronic anemia varies between 30% and 70% and rheumatoid nodules have been reported from 2.1% to 38.2% [1, 13].

Because the main objective of the study was to identify the possible association of anti-CCP or anti-MCV autoantibodies with ExRA, we examined primarily all the patients that had one or more ExRA and subsequently, those patients were with a specific ExRA. We did not observe an association between the presence of ExRA as total group and positivity for anti-CCP. Similar results were reported by Sghiri et al. who did not observe association between ExRA and anti-CCP antibodies [10]. Our data differ from observations made by Salinas et al. [14] who described a significant association between anti-CCP and ExRA. On the other side, Turesson et al. observed an association between anti-CCP and ExRA but only in those patients with severe ExRA, whereas this association does not persisted when ExRA was evaluated in general [7]. Unfortunately, a limitation in our study was that the number of patients with severe ExRA was too small to allow statistical comparisons. Regarding the SS, we did not observe an association between SS and positivity for anti-CCP or anti-MCV antibodies. While some groups designate the SS associated to RA as a “secondary” SS, this syndrome could be considered as an overlap disease more than an ExRA [15]. Barcelos et al. identified that patients with secondary SS had higher frequency of anti-CCP antibodies [16] although their sample was small and therefore is limited in its appropriateness to generalize their results. We have recently informed the results of a case-control study performed obtained from a different population where we observed a significant association between positivity for anti-CCP antibodies and the presence of interstitial lung disease associated to RA (ILD-RA) [17]. Nevertheless, in this present study, we were limited to evaluate this association because the low prevalence observed of ILD-RA.

We did not observe an association between anti-MCV and ExRA as total group. Our data are similar to those described by Sghiri et al. [10]. Although, as described above, we have found a weak but significant association between positivity for this autoantibody and rheumatoid nodules, finding that requires to be reproduced by other studies. An association was observed between the nonutilization of MTX and ExRA, although this association was not observed with dose or anti-TNF agents. Mikuls et al. identified that during the course of treatment, around a half of their patients experienced a decrease in RF or anti-CCP levels [18]. These data are in concordance with our findings related to lower frequency of ExRA in MTX users. On the other side Nicaise Roland et al. have described that patients with RA treated with infliximab may have a decrease in anti-CCP and anti-MCV levels with the therapy [19]. We did not identified statistical differences on the frequency of ExRA in users of anti-TNF agents, nevertheless, a clear limitation for our analysis is the small proportion of patients that were treated with these agents.

Our study has several limitations, first because our study was designed to include consecutive patients with RA tested for presence of ExRA and although we evaluated 225 patients, we were unable to identify a significant number of severe ExRA, therefore, we did not test associations in these subgroups. Nevertheless, we identified a significant number of patients with nonsevere ExRA and found no associations except for rheumatoid nodules. Another limitation inherent to this cross-sectional design is that we only tested for anti-CCP and anti-MCV antibodies in one occasion, being necessary in further studies to test these auto-antibodies several times to identify variations in their titers. Hence, further longitudinal studies are required to test if patients with RA with higher titers for these autoantibodies will develop a higher rate of ExRA in the long-run. Another limitation of our study is the long disease duration observed in some of our patients, in this case is expected that the age of our patients at the time of the study is closely related with RA duration. Therefore we observed in the multivariate analysis that disease duration is relevant confounder in the final model.

Relevantly, in the best of our knowledge this is the first study performed in Mexican patients that test the association between anti-CCP or anti-MCV anti-bodies with ExRA, and also is one of the few studies where patients are obtained from a secondary-care center; this last characteristic constitutes an advantage compared with studies performed in tertiary-care centers where ExRA is expected be more severe and are more likely to have referral bias.

In conclusion, we identified that 42% of our patients with RA have extra-articular manifestations. Variables associated with ExRA were age, disease duration, DAS-28 score, and HAQ-DI score. This study did not observe an association between ExRA as total group and the positivity or titers of anti-CCP or anti-MCV antibodies. Although a weak association was observed between positivity for these autoantibodies and rheumatoid nodules. Further studies with more severe ExRA should be performed to identify if these autoantibodies are associated with these specific manifestations.

## Figures and Tables

**Table 1:** Specific extra-articular manifestations evaluated in patients with RA.

Extra-articular manifestation	Criteria
(1) Pericarditis	Clinical judgment and confirmed by echocardiography

(2) Pleuritis	Clinical judgment and thorax radiographs

(3) Felty's syndrome	Clinical evidence confirmed by ultrasound and neutropenia <1.8 × 10^9^ described in at least 2 occasions

(4) Major cutaneous vasculitis	Clinical judgment confirmed by biopsy

(5) Neuropathy	Clinical judgment and positive results poly/mononeuropathy at electromyography

(6) Scleritis, epiescleritis, uveitis, or retinal vasculitis	Identified by an specialist and if required a biopsy was performed

(7) Glomerulonephritis	Corroborated by nephrologist and if required a renal biopsy was performed

(8) Vasculitis involving other organs	Identified by an specialist and if required a biopsy was performed

(9) Amyloidosis	Clinical judgment and positive biopsy if required

(10) Keratoncunjunctivitis sicca	Clinical judgment: (a) positive Rose-Bengal staining and (b) positive Schirmer's test <5 mm/5 mn

(11) Xerostomia	Clinical judgment and abnormal sialometry and if suspected minor salivary gland biopsy with lymphocytic infiltrate

(12) Secondary Sjögren's syndrome	Diagnosed by two of the following criteria (a) keratonconjunctivitis sicca, (b) xerostomia, and (c) positivity for anti-Ro or anti-La antibodies

(13) Bronchiolitis obliterans	Clinical judgment by pulmonologist

(14) Organizing Pneumonia	Clinical judgment by pulmonologist

(15) Pulmonary fibrosis	Clinical judgment by pulmonologist plus restrictive pattern in lung function test and confirmed by positive findings in high-resolution computed tomography of the lung

(16) Cervical myelopathy	Clinical judgment and radiograph showing increased in atlantoaxial distance

(17) Subcutaneous rheumatoid nodules	Clinical judgment and biopsy if required

(18) Chronic anemia	Diagnosed if a hemoglobin <11 g/dL was observed in the last 6 months before the evaluation and other causes of anemia were excluded

**Table 2:** Selected characteristics in patients with rheumatoid arthritis.

Characteristic	RA *n* = 225
Female, *n* (%)	207 (92.0)
Age, years	52.47 ± 10.96
Alcohol consumption*, *n* (%)	28 (12.4)
Smoke exposure, *n* (%)	64 (28.4)
RA characteristics	
Disease duration (years), mean ± SD	8.97 ± 8.32
DAS28, mean ± SD	5.04 ± 1.36
HAQ-Di score (units)	0.84 ± 0.67
Global functional status III-IV, *n* (%)	43 (19.1)
Steinbrocker stage in hands III or IV *n* (%)	94 (41.8)
ExRA, *n* (%)	95 (42.2)
Extra-articular manifestations	
Sjögren's Syndrome, *n* (%)	58 (25.8)
Chronic anemia, *n* (%)	33 (14.7)
Rheumatoid nodules, *n* (%)	23 (10.2)
Peripheral neuropathy, *n* (%)	12 (5.3)
Pulmonary fibrosis, *n* (%)	6 (2.7)
Raynaud's phenomenon, *n* (%)	3 (1.3)
Felty's Syndrome, *n* (%)	2 (0.9)
Thrombosis, *n* (%)	2 (0.9)
Scleritis, *n* (%)	1 (0.4)
Intestinal vasculitis, *n* (%)	1 (0.4)
Laboratory findings	
ESR, mm/h	29.22 ± 11.90
CRP, mg/L	20.85 ± 30.83
Positive RF**, *n* = 204 (%)	135 (66.2)
RF titres, IU/mL	119.11 ± 237.99
Positive anti-CCP	155 (68.9)
Anti-CCP titres, IU/mL	72.44 ± 88.22
Positive anti-MCV	157 (69.7)
Anti-MCV titres, IU/mL	156.85 ± 183.50
(+) anti-CCP and (+) anti-MCV, *n* = 225	140 (62.2)
(+) anti-CCP, (+) anti-MCV and (+) RF, *n* = 204	110 (53.9)
Treatment	
Synthetic DMARDs, *n* (%)	206 (92.6)
MTX users, *n* (%)	180 (80.0)
Anti-TNF*α* agents, *n* (%)	19 (8.4)
Corticosteroids, *n* (%)	214 (95.1)
Prednisone (current doses), mg/day	5.24 ± 2.06

RA: rheumatoid arthritis; *Alcohol consumption: defined as consume of at least one alcoholic beverage on a daily basis in the last year. DAS28: disease activity score; HAQ-Di: Health Assessment Questionnaire-Disability Index; ExRA: rheumatoid arthritis with extra-articular manifestations; ESR: erythrocyte sedimentation rate; **RF: rheumatoid factor (only assessed in two thousand four patients); CRP: C-Reactive Protein; anti-CCP: anti-cyclic citrullinated peptide antibodies; anti-MCV: anti-mutated citrullinated vimentin, DMARDs: disease-modifying antirheumatic drugs.

Qualitative variables are expressed as frequencies (%); quantitative variables are expressed as means and standard deviation.

**Table 3:** Comparisons of characteristics between patients with ExRA and without extra-articular manifestations (RA w/out).

Clinical characteristics	RA w/out *n* = 130	ExRA *n* = 95	*P*
Female, *n* (%)	116 (89.2)	91 (95.8)	0.73
Age, years	50.75 ± 10.92	54.82 ± 10.62	0.006
Alcohol consumption, *n* (%)	21 (16.2)	7 (7.4)	0.49
Smoke exposure, *n* (%)	30 (23.1)	34 (35.8)	0.04
Disease duration, years	7.04 ± 6.57	11.60 ± 9.69	<0.001
DAS28	5.03 ± 1.34	5.05 ± 1.39	0.91
Tender joint count	4.28 ± 4.91	7.40 ± 7.18	0.004
Swollen joint count	6.42 ± 6.89	7.47 ± 8.08	0.43
VAS global	42.71 ± 26.48	42.80 ± 28.70	0.16
HAQ-Di score (units)	0.89 ± 0.66	0.77 ± 0.69	0.18
Global functioning status III-IV, *n* (%)	23 (17.7)	20 (21)	0.53
Radiological Stage III or IV in hands, *n* (%)	45 (34.7)	49 (51.6)	0.01
ESR, mm/h	29.40 ± 11.56	28.97 ± 12.40	0.79
CRP, mg/L	15.43 ± 19.84	27.31 ± 39.36	0.01
RF, IU/mL	119.07 ± 252.69	119.16 ± 220.03	0.99
Anti-CCP, IU/mL	67.99 ± 74.14	78.62 ± 104.86	0.40
Anti-MCV, IU/mL	155.60 ± 179.03	158.58 ± 190.44	0.91
MTX users, *n* (%)	112 (86.2)	68 (71.6)	0.007
MTX dose, mean ± SD	7.33 ± 4.14	6.37 ± 5.16	0.12
Anti-TNF agents users, *n* (%)	9 (6.9)	10 (10.5)	0.34

RA w/out: rheumatoid arthritis (RA) without extra-articular manifestations; ExRA: RA with extra-articular manifestations; DAS28: disease activity score; HAQ-Di: Health Assessment Questionnaire-Disability Index; ESR: erythrocyte sedimentation rate; RF: rheumatoid factor; CRP, C-Reactive Protein; anti-CCP: anti-cyclic citrullinated peptide antibodies; anti-MCV: anti-mutated citrullinated vimentin, DMARDs: disease-modifying antirheumatic drugs. Qualitative variables are expressed as frequencies (%); quantitative variables are expressed as mean and standard deviation. Comparisons between proportions were compared with Chi-square or Fisher exact test (when required). Comparisons between means were evaluated with Student's *t*-test for independent samples.

**Table 4:** Comparisons of frequencies of ExRA and specific extra-articular manifestations according to the findings of positive or negative anti-CCP or anti-MCV.

Characteristics	(−) RF *n* = 69	(+) RF *n* = 135	*P*	(−) anti-CCP *n* = 70	(+) anti-CCP *n* = 155	*P*	(−) anti-MCV *n* = 68	(+) anti-MCV *n* = 157	*P*
Presence of ExRA, *n* (%)	30 (43.5)	57 (42.2)	0.86	27 (38.6)	68 (43.9)	0.46	26 (38.2)	69 (43.9)	0.43
Specific manifestations									
Sjögren syndrome, *n* (%)	23 (33.3)	29 (21.5)	0.07	18 (25.7)	40 (25.8)	1.00	16 (23.5)	42 (26.8)	0.61
Chronic anemia, *n* (%)	9 (13.0)	23 (17.0)	0.46	7 (10.0)	26 (16.8)	0.18	9 (13.2)	24 (15.3)	0.69
Rheumatoid nodules, *n* (%)	2 (2.9)	19 (14.1)	0.01	3 (4.3)	20 (12.9)	0.048	2 (2.9)	21 (13.4)	0.02
P. neuropathy, *n* (%)	6 (8.7)	5 (3.7)	0.19	5 (7.1)	7 (4.5)	0.52	4 (5.9)	8 (5.1)	0.76

The cut-off value to be considered anti-CCP as positive was ≥5 IU/mL, for anti-MCV, the levels considered as positive were ≥20 IU/mL. RA: rheumatoid arthritis; ExRA: extra-articular manifestations; anti-CCP: anti-cyclic citrullinated peptide antibodies; anti-MCV: anti-mutated citrullinated vimentin antibodies, P. neuropathy (peripheral neuropathy). Qualitative variables are expressed as frequencies (%). Comparisons between proportions were compared with Fisher exact test.

**Table 5:** Comparison in characteristics between specific ExRA manifestations and w/out these manifestations.

Clinical characteristics	Sjögren's syndrome		*P*	Chronic anemia		*P*	Rheumatoid nodules		*P*	Peripheral neuropathy		*P*
	Yes *n* = 58	No *n* = 167		Yes *n* = 33	No *n* = 192		Yes *n* = 23	No *n* = 202		Yes *n* = 12	No *n* = 213	
Female, *n* (%)	56 (96.6)	151 (90.4)	0.17	32 (97.0)	175 (91.1)	0.48	22 (95.7)	185 (91.6)	0.70	12 (100)	195 (91.5)	0.29
Age, years	51.0 ± 11	56.7 ± 9.1	0.001	52.9 ± 11	50.0 ± 12	0.17	52.1 ± 11	55.1 ± 12	0.22	52.1 ± 11	58.0 ± 7.8	0.07
Disease duration, years	7.9 ± 7.5	11.9 ± 9.7	0.005	9.0 ± 8.4	8.5 ± 7.6	0.74	8.1 ± 7.7	16.2 ± 9.7	0.001	8.8 ± 8.3	12.0 ± 7.4	0.19
Year of diagnosis before 2000	20 (34.5)	30 (18.0)	0.009	6 (18.2)	44 (22.9)	0.55	10 (43.5)	40 (19.8)	0.010	4 (33.3)	46 (21.6)	0.31
DAS28	5.1 ± 1.3	4.9 ± 1.3	0.29	4.9 ± 1.3	5.6 ± 1.2	0.005	5.03 ± 1.3	5.11 ± 1.3	0.79	5.1 ± 1.3	4.6 ± 1.5	0.21
HAQ-Di score (units)	0.88 ± 0.67	0.72 ± 0.66	0.11	0.9 ± 0.7	0.8 ± 0.7	0.81	0.9 ± 0.7	0.7 ± 0.6	0.29	0.9 ± 0.7	0.7 ± 0.6	0.49
Radiological III-IV hands, *n* (%)	28 (48.3)	66 (39.5)	0.24	17 (51.5)	77 (40.1)	0.22	16 (69.6)	78 (38.6)	0.004	7 (58.3)	87 (40.8)	0.23
ESR, mm/h	30.0 ± 11.9	26.8 ± 11.6	0.08	28.9 ± 12	31.1 ± 14	0.33	29.0 ± 11.9	30.8 ± 11.7	0.51	29.5 ± 12	23.5 ± 10	0.09
CRP, mg/L	21.5 ± 32.1	19.1 ± 27.0	0.62	16.8 ± 22.2	40.7 ± 52.5	0.015	19.6 ± 28.5	31.1 ± 45.1	0.26	21.6 ± 32	9.2 ± 9	0.19
(+) RF*, *n* (%)	29 (55.8)	106 (69.7)	0.07	23 (71.9)	112 (65.1)	0.46	19 (90.5)	116 (63.4)	0.01	5 (45.5)	130 (67.4)	0.19
(+) Anti-CCP, *n* (%)	40 (69.0)	115 (68.9)	0.99	26 (78.8)	129 (67.2)	0.18	20 (87.0)	135 (66.8)	0.048	7 (58.3)	148 (69.5)	0.52
(+) Anti-MCV, *n* (%)	42 (72.4)	115 (68.9)	0.61	24 (72.7)	133 (69.3)	0.69	21 (91.3)	136 (67.3)	0.02	8 (66.7)	149 (70.0)	0.76
Chloroquine users, *n* (%)	14 (24.1)	17 (10.2)	0.008	4 (12.1)	27 (14.1)	1.00	4 (17.4)	27 (13.4)	0.53	1 (8.3)	30 (14.1)	1.00
MTX users, *n* (%)	41 (70.7)	139 (83.2)	0.04	26 (78.8)	154 (80.2)	0.85	12 (52.2)	168 (83.2)	0.001	8 (66.7)	172 (80.8)	0.26
MTX doses, mean ± SD	7.2 ± 4.4	6.2 ± 5.0	0.15	6.8 ± 4.4	7.6 ± 5.5	0.46	7.0 ± 4.4	5.7 ± 6.0	0.32	7.0 ± 4.6	4.8 ± 4.4	0.10
Azathioprine users, *n* (%)	6 (10.3)	13 (7.8)	0.55	5 (15.2)	14 (7.3)	0.17	9 (39.1)	10 (5.0)	<0.001	2 (16.7)	17 (8.0)	0.27
Anti-TNF agents, users, *n* (%)	6 (10.3)	13 (7.8)	0.59	2 (6.1)	17 (8.9)	1.00	4 (17.4)	15 (7.4)	0.11	0 (0.0)	19 (8.9)	0.61

RA: rheumatoid arthritis; DAS28: disease activity score; HAQ-Di: Health Assessment Questionnaire-Disability Index; ExRA: rheumatoid arthritis with extra-articular manifestations; ESR: erythrocyte sedimentation rate; CRP: C-Reactive Protein; *RF: rheumatoid factor (only assessed in two thousand four patients); anti-CCP: anti-cyclic citrullinated peptide antibodies; anti-MCV: anti-mutated citrullinated vimentin, DMARDs: disease-modifying antirheumatic drugs. Qualitative variables are expressed as frequencies (%); quantitative variables are expressed as means and standard deviation. Comparisons between proportions were made with Chi-square (or Fisher exact test if required). Comparisons between means were made using Student's *t*-test.

**Table 6:** Multivariate logistic regression testing for variables associated with ExRA.

Criterion predictor	Method Enter			Method forward stepwise		
	OR	95% CI	*P*	OR	95% CI	*P*
Age, years	1.08	1.02–1.13	0.002	1.05	1.01–1.10	0.015
Disease duration, years	1.13	1.04–1.23	0.002	1.09	1.02–1.16	0.007
Smoke exposure	0.70	0.25–2.00	0.41	Not in the model	—	—
DAS-28	2.24	1.42–3.52	<0.001	1.86	1.27–2.73	0.002
Functional Impairment (HAQ-Di)	5.62	1.78–17.75	0.003	4.46	1.51–13.14	0.007
CRP mg/L	1.02	1.00–1.05	0.025	Not in the model	—	—
ESR mm/hr	0.95	0.90–0.99	0.02	Not in the model	—	—
RF IU/mL	1.27	0.45–3.54	0.64	Not in the model	—	—
Anti-CCP IU/mL	1.00	0.99–1.01	0.84	Not in the model	—	—
Anti-MCV IU/mL	1.00	0.99–1.002	0.63	Not in the model	—	—
Anti-TNF agents	1.63	0.36–7.50	0.53	Not in the model	—	—

DAS28: disease activity score 28-joints assessed; HAQ-Di: Health Assessment Questionnaire-Disability Index; MTX: methotrexate; Anti-CCP: anti-cyclic citrullinated peptide antibodies, OR: odds ratio, 95% CI, 95% confidential intervals.
